# Surgical Outcomes and Patient Satisfaction With the Low-Cost, Semi-Rigid Shah Penile Prosthesis: A boon to the Developing Countries

**DOI:** 10.1016/j.esxm.2021.100399

**Published:** 2021-07-16

**Authors:** Pramod Krishnappa, Amit Tripathi, Rupin Shah

**Affiliations:** 1 Division of Andrology, Department of Urology, NU Hospitals, Bangalore, India; 2 Division of Andrology, Department of Urology, Lilavati Hospital and Research Centre, Mumbai, India

**Keywords:** Shah Penile Prosthesis, Semirigid Penile Prosthesis, Erectile Dysfunction, EDITS, Residual Penile Tumescence

## Abstract

**Introduction:**

In developing countries most patients with refractory erectile dysfunction cannot afford a penile prosthesis (PP) due to its cost and non-coverage by insurance companies.

**Aim:**

To assess the patient satisfaction outcomes with a novel, low-cost, semi-rigid PP.

**Methods:**

52 patients who had received the Shah semi-rigid PP between January 2013 and December 2018 were included in this bidirectional study. Patient demographics including age, etiology, body mass index, length of PP received and post-operative complications were recorded. Patient satisfaction with the PP was evaluated using the modified Erectile Dysfunction Inventory of Treatment Satisfaction (EDITS) Questionnaire.

**Main outcome measures:**

The primary outcome measures were overall satisfaction, total EDITS and mean EDITS score. The secondary outcome measures were residual penile tumescence, ease of concealment and post-operative complications.

**Results:**

The mean age of the patients was 38.79 years (25–68). Overall satisfaction (EDITS Q-1) of 4 (0–4) was reported by 84.62% (44/52) of patients. There was no significant difference (*P* > .7) in the total EDITS and overall satisfaction based on various etiological factors. The mean EDITS scores (0–100) were 95.67 ± 10.76, 95.53 ± 8.46 and 91.72 ± 22.42 in 52 patients with BMI <25, 25–29.9 and >30 kg/m2 respectively. During sexual arousal after PP implantation, 26 (50%), 17 (32.7%) and 9 (17.3%) patients noted “good”, “some” or “no” residual penile tumescence respectively. 47 (90.4%), 4 (7.7%) and 1 (1.9%) patients reported “good”, “fair” and “poor” concealment respectively. In the prospective group, major and minor post-operative complications were seen in 10.7% (3/28) and 21.4% (6/28) of patients respectively.

**Conclusion:**

The semi-rigid Shah PP is a safe, effective and affordable option to treat patients with refractory ED. The ability to remove 1 or both sleeves in the Shah PP helps achieve a good fit with a small inventory. **Krishnappa P, Tripathi A, Shah R. Surgical Outcomes and Patient Satisfaction With the Low-Cost, Semi-Rigid Shah Penile Prosthesis: A boon to the Developing Countries. Sex Med 2021;9:100399.**

## INTRODUCTION

Penile prosthesis (PP) implantation has been the mainstay of treatment in patients presenting with refractory erectile dysfunction (ED). However, despite its efficacy only a small proportion of men who would benefit from a PP get operated. A recent systematic review article highlighted the fact that it takes a mean duration of 72.2 months (range 40.3–102) and 56 months (range 35.8–144) for patients to seek semi-rigid PP (SPP) and inflatable PP (IPP) respectively.^1^

There are several reasons for under-utilization of PP. Physicians tend to perceive ED as not a serious condition; this belief affects General Practitioners’ prescribing practice and becomes a barrier preventing many ED patients from being referred for further treatment when non-surgical therapies fail.^2^

Another major limiting factor in many parts of the world is cost. The relatively high costs of the globally available SPP and IPP have resulted in PP being inaccessible to majority of the ED patients in developing countries.

**Figure 1:**
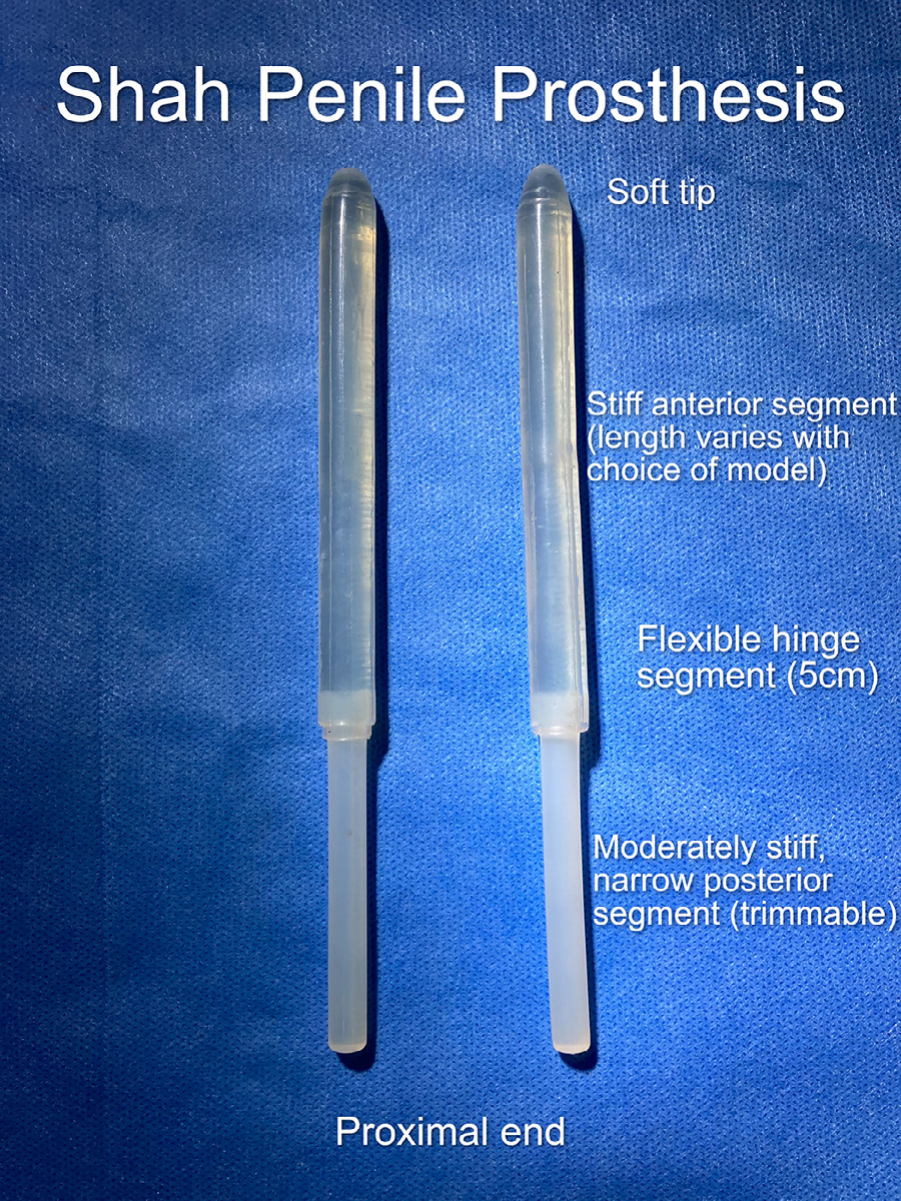
Differential rigidity of Shah penile prosthesis.

The Shah PP (G.Surgiwear, Rasoolpur, UP, India) is a low cost, non-inflatable (semi-rigid), differential rigidity (flexible) silicone implant that provides rigidity through a stiff anterior zone and concealment through a soft, central flexible hinge zone^3^ (Figure 1).

**Figure 2:**
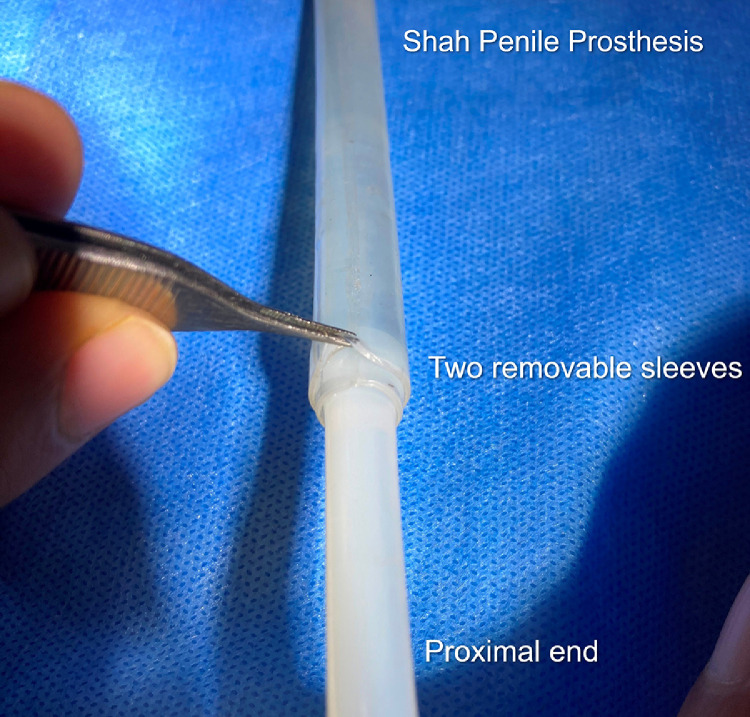
2 removable sleeves of Shah penile prosthesis.

It comes in different models that have varying lengths of the anterior stiff zone so as to achieve an optimal fit in penises of different lengths A unique feature is the presence of 2 removable sleeves (Figure 2) which enable adjustment of the diameter of the PP from 13 mm to 11 mm to 9 mm (in models WH9 and WH 11), and from 15 mm to 13 mm to 11 mm (in models WH13 and WH15); this allows adjustment to the most appropriate diameter to obtain a snug fit in the corpora, without the need for a large inventory.

The Shah PP is the most preferred and widely used PP in India but is not recognised in global reviews^4^ on PP except for a brief mention of the name of Shah PP in a review paper from Chung E.^5^ The aim of this study is to test the hypothesis that a low cost, non-inflatable implant can provide high satisfaction rates in men with severe ED. This is the first study to document the short-term and long-term outcomes of Shah PP in a large number of patients.

## MATERIALS AND METHODS

This was a bidirectional (retrospective and prospective) study which included refractory ED patients who received Shah PP by a single surgeon (RS) from Jan 2013 to Dec 2018. The retrospective group was followed up telephonically between August 2018 and December 2018 that is, 2–5 years after their surgery. Prospective patients were followed up at 1 week, 4 weeks and 12 weeks after surgery, and the final outcome and EDITS score were assessed 6 months after surgery. If the patient was resident of the city, the follow-up was in person. If the patient was from out-of-town then the follow-up was telephonic, and an in-person follow-up was done only if there was any reported problem or complication. Institutional ethics committee approval was obtained (ECR/606/Inst/MH/2014/RR-17). Consent waiver in retrospective cases was given by the Institutional ethics committee and informed written consents were obtained in prospective cases.

All the patients had failed to respond to multiple doses of oral phosphodiesterase type 5 inhibitor and to full doses of intra-cavernosal injections (ICI) with bimix and trimix. They received the Shah PP through the standard penoscrotal approach. The model to be implanted was selected on the basis of the stretched penile length (SPL) from symphysis pubis to mid-glans (Table 1).This is very important to ensure optimal positioning of the hinge segment and attain a proper balance between rigidity and concealment.

**Table 1:** Selection of Shah Penile Prosthesis based on stretched penile length

Stretched penile length*	Choice of PP (model)	Length of stiff anterior segment of the PP	Diameter of prosthesis (2 removable sleeves)
<9 cm	OH 01	Uniform rigidity, no hinge segment	13 mm→11 mm→9 mm
9–10 cm	WH 09	7 cm	13 mm→11 mm→9 mm
11–12 cm	WH 11	9 cm	13 mm→11 mm→9 mm
13–14 cm	WH 13	11 cm	15 mm→13 mm→11 mm
>15 cm	WH 15	13 cm	15 mm→13 mm→11 mm

⁎ From symphysis pubis to mid-glans

The largest diameter that could be comfortably inserted was used, and 1 or both sleeves were removed, if necessary, to get the best fit. The length of the PP was adjusted to the measured intracorporal length by trimming the proximal segment and using rear tip extenders (RTE) as needed. The patients received intravenous cefuroxime and gentamycin on the morning and evening of surgery, and the next day morning before discharge, followed by oral amoxicillin- clavulanate for next 5 days. Guidelines^6^ do not recommend routine post-operative prophylactic antibiotics, but we prefer to give oral antibiotics for 5 days.^7^

Foley catheter was removed on 1st post-operative day. Sexual intercourse was permitted after 6–8 weeks after the PP implantation.

### Erectile Dysfunction Inventory of Treatment Satisfaction (EDITS) Questionnaire

Patient satisfaction was evaluated using modified EDITS which is a standardized assessment tool adapted to PP devices. EDITS is a validated questionnaire developed by Althof et al^8^ to assess satisfaction following medical ED treatment. This was later modified by Levine et al^9^ to assess satisfaction following PP implantation. The questions addressed overall patient satisfaction, the degree to which PP met patient expectations, the likelihood of continued use, ease of use of the device, confidence in ability to engage in sexual activity, patient assessment of partner satisfaction, patient assessment of partner feelings about continuing use of the prosthesis, rigidity, and appearance. We added 2 additional unvalidated questions on “ease of concealment” and “residual penile tumescence following PP implantation.” The modified EDITS patient's survey form (Supplementary file) has 11 questions; each question has a minimum score of zero and maximum of 4. Hence the maximal “Total EDITS” is 44. “Mean EDITS” = Total EDITS divided by 11. “Mean EDITS score” = mean EDITS multiplied by 25. Questionnaire was translated to Marathi and Hindi which are the most common languages spoken by the study population besides English.

### Statistical Analysis

Data was analyzed using Statistical Package for Social Sciences V15.0 package (Statistical Package for Social Sciences Inc, Chicago, Illinois). Continuous variables were expressed as Mean and SD for normal data and median and range for non-normal data. Comparisons of continuous variables among groups were carried out by Student's unpaired t test for normal data and Mann Whitney U test for non-normal data. Categorical variables were expressed as frequencies and percentages and were compared between 2 groups using the Fisher exact test or Chi square test. 1-way ANOVA was used to make comparisons between the means of 3 or more groups of data. All statistical tests were 2 tailed. Alpha (α) level of significance was taken as *P* ≤ 0.05.

## RESULTS

The retrospective group comprised of 34 patients operated between January 2013 and December 2016. The prospective group comprised of 34 patients operated between January 2017 and December 2018. 8 patients (8/34) from the retrospective group could not be reached for follow-up. In the prospective group, 6 were not available for post-op follow-up; hence post-op complications are reported in 28 prospective patients. Of these 28 patients in the prospective group, 2 were explanted during the immediate post-operative period, and hence 26 in prospective group were assessed for satisfaction at 6 months. So, finally 52 (26 + 26) patients were assessed for their satisfaction and 28 patients were assessed for complications. The patient demographics and mean age distribution as per the etiology has been presented in Table 2.

**Table 2:** Mean age distribution of Shah penile prosthesis patients as per the etiology

Etiology	N (%)	Mean age (y)
Atherosclerotic vasculogenic	13 (25)	46.38 ± 11.62
Traumatic vasculogenic	5 (9.6)	33.20 ± 7.92
Ischemic priapism	7 (13.5)	34.71 ± 6.75
Peyronie's disease	3 (5.8)	42.67 ± 12.74
Miscellaneous (unknown and rare etiologies)	24 (46.2%)	36.54 ± 8.93
Total	52 (100)	

The mean age of the patients was 38.79 years (25–68) and majority (86.5%) of our patients were less than 50 years age.

### Modified EDITS Questionnaire outcomes

1. Overall satisfaction & Total EDITS:

The first question of the modified EDITS survey assessed the overall satisfaction (0–4). The overall satisfaction of 4/4 was found in 84.62 % of patients (44/52). There was no significant difference (*P* > .7) in the total EDITS and overall satisfaction based on various etiological factors (Table 3).

2. Mean EDITS & Mean EDITS score:

**Table 3:** Distribution of overall satisfaction and Total EDITS in various etiologies

Etiology	N	Overall satisfaction (0–4) [mean]	Total EDITS (0–44) [mean]
Atherosclerotic vasculogenic	13	3.54 ± 1.13	40.00 ± 8.38
Traumatic vasculogenic	5	3.80 ± 0.45	42.40 ± 3.58
Ischemic priapism	7	3.86 ± 0.38	42.86 ± 2.27
Peyronie's disease	3	4.00 ± 0.00	44.00 ± 0.00
Miscellaneous (unknown and rare etiologies)	24	3.79 ± 0.66	42.08 ± 4.38
Comparison among 4 groups (1 way ANOVA)		F = 0.4,NS, *P* = .8	F = 0.6,NS, *P* = .7

Majority of the patients were satisfied with the Shah PP irrespective of age, BMI and PP size. The mean EDITS (0–4) was 3.93 ± 0.25, 3.84 ± 0.35, 3.40 ± 0.99 in age groups <30 years, 30–50 years and >50 years (*P* = not significant).

Mean BMI of the patients was 26.29(18.8–36.1) kg/m^2^. A subgroup analysis of mean EDITS and mean EDITS score across various BMI was assessed and found to have no statistical significance (p 0.7). The mean EDITS score (0–100) were 95.67 ± 10.76, 95.53 ± 8.46 and 91.72 ± 22.42 (p 0.7) in patients with BMI <25, 25–29.9 and >30 kg/m^2^respectively (Table 4).

**Table 4:** Effect of BMI on overall satisfaction score and mean EDITS score

BMI	N	Overall satisfaction (0–4)	Mean EDITS (0–4)	Mean EDITS score (0–100)
<25	19	3.74 ± 0.73	3.83 ± 0.43	95.67 ± 10.76
25–29.9	25	3.84 ± 0.37	3.82 ± 0.34	95.53 ± 8.46
≥30	8	3.50 ± 1.41	3.67 ± 0.90	91.72 ± 22.42
1 way ANOVA		F = 0.6,NS,P = 0.5	F = 0.3,NS,*P* = .7	F = 0.3,NS,*P* = .7

5 different sizes of Shah PP were used: OH01, WH09, WH11, WH13 and WH15. OH stands for “withOut Hinge” and is used when the SPL is <9 cm. WH stands for “With Hinge” while the model number refers to the matching SPL (Table 1). For statistical ease, 3 subdivisions were done as follows: small (OH01 and WH09), medium (WH11) and large (WH13 and WH15). Majority (53.8%) of the patients (28/52) received medium sized PP followed by small sized PP in 13/52 (25%) patients and large size PP in 11 patients (21.2%). Only 1 patient received OH01 (without hinge) as the penis was short. The mean EDITS score was 91.94 ± 17.75, 96.72 ± 8.29 and 94.20 ± 12.54 in patients with small, medium and large size PP group respectively (Table 5).

**Table 5:** Effect of implant size on mean EDITS score and overall satisfaction

Types	N (%)	Mean EDITS (0-4)	Mean EDITS score (0-100)	Overall satisfaction (0–4)
Small (OH01 and WH09)	13 (25)	3.68 ± 0.71	91.94 ± 17.75	3.54 ± 1.12
Medium (WH11)	28 (53.8)	3.87 ± 0.33	96.72 ± 8.29	3.89 ± 0.32
Large (WH13 and WH 15)	11 (21.2)	3.77 ± 0.50	94.20 ± 12.54	3.64 ± 0.92
1 way ANOVA		F = 0.7, NS, *P* = .5	F = 0.7, NS, *P* = .5	F = 1.2, NS, *P* = .3

### Residual Penile Tumescence

Despite having most of the corpora occupied by the prosthesis, residual penile tumescence during arousal was noted in the majority of patients. 26 patients (50%) had “good” residual tumescence, 17 patients (32.7%) reported “some” residual tumescence and 9 patients (17.3%) had no residual tumescence. The mean EDITS score (and overall satisfaction) of patients with “good”, “some” and “nil” residual tumescence were 99.9 ± 0.49 (4), 93.04 ± 12.92 (3.72 ± 0.52) and 84.50 ± 19.58 (3.38 ± 0.78) respectively (*P* = .005).

### Ease of Concealment

47 patients (90.4%) reported no problem with concealment. 4 patients (7.7%) needed some modification of their undergarments to conceal the PP. Only 1 patient reported significant difficulty with concealment and got the PP explanted.

### Complications

During the prospective follow-up of 28 cases, major post-operative complications were found in 10.7% (3/28) of patients. Urethral perforation in 1 patient and deep infection in another patient required explantation of the PP. Urethral perforation was not recognized intra-operatively. Patient had urinary retention on 2nd post-op day after urethral catheter removal. On examination, the tips of the implants could be seen through the meatus and both implants were pulled out; the patient was successfully re-implanted 3 months later.

The patient with deep infection presented 10 days after surgery. He was initially treated with intravenous antibiotics but required exploration after 2 days of antibiotics as the wound worsened. On exploration, pus was drained out from both corpora cavernosa and PP was removed. The bacteria isolated from pus was Staphylococcus aureus

The third patient presented with impending erosion of 1 implant at the glans. This was successfully corrected by shortening the implant to the level of the coronal sulcus, and then fixing it in place with sutures passed through the tunica and the implant.

Minor complications were seen in 21.4% (6/28) of the patients which included penile edema in 4, prolonged penile pain in 1 and superficial infection in 1. All 6 patients were managed conservatively.

## DISCUSSION

The quest for an affordable PP for Indian patients eventually culminated in the development of Shah PP. The prototype was first implanted in 1996 and it evolved into its present form over the next 2 years.^3^ The Shah PP was initially priced at INR 6000 and currently costs INR 23,000 (300 USD). The Implant cost of Tactra (Boston Scientific, Marlborough, MA, USA), AMS Ambicor (Boston Scientific, Marlborough, MA, USA) and AMS 700 CX (Boston Scientific, Marlborough, MA, USA) would be approximately 1800 USD, 6700 USD and 10500 USD respectively in India. No insurance companies cover any penile implant charges in India.

This is the first study to report the short-term and long-term outcomes of this low cost implant, although it is being widely used in India since over 2 decades. Each set of Shah PP contains 1 pair of PP, 3 pairs of RTE (1, 2 and 3 cm) and 1 plastic adapter (to help trimming of proximal part of PP). 4 hinged models are available, having different lengths of the stiff segment and different diameters (Table 1).For instance, Shah PP “WH13” is suitable for SPL 13–14 cm;it has a diameter of 15 mm (adjustable to 13 mm by removing the outer sleeve or to 11 mm by removing both sleeves).

Currently, IPP constitutes upto 90% of the PP done in the USA due to insurance coverage and better cosmetic results.^10^ However, this is not the case in most developing countries, including India, as the cost of the IPP is too expensive and not covered under insurance. Hence, the SPP continues to be the major workhorse of most Andrologists in developing countries, while in developed countries its use is restricted to those who prefer a simple device or have impaired manual dexterity or for a salvage procedure (following infection or longstanding priapism).^11^

It is generally assumed that younger patients do not need a PP. However, we had 11 patients less than 30 years of age that needed the PP to become sexually potent. The high proportion of young patients in our study is due to Indian socio-cultural attitudes that lead older men with ED to reject surgery as a solution for their problem and restrict their therapeutic choices to phosphodiesterase type 5 inhibitor or ICI. Thus, as is shown in Table 2, not one of our patients was operated for post-radical prostatectomy ED, which is one of the commonest indications for PP in the West.^1^

In our country the majority who opt for surgery are young men who want to get married, or are married and unable to consummate their marriage, and have severe organic ED. Many Indian men do not have premarital intercourse and some consider masturbation to be harmful. Hence, they are unaware of their ED till they get married and many actually end up with a divorce due to non-consummation of the marriage.

Many studies have confirmed high satisfaction rates with IPP^12^^,^^13^ while SPP has been reported to have lower satisfaction rates than IPP,^14^ but in our study 84.6% of the patients were highly satisfied with Shah SPP with mean EDITS score of 95. Possible reasons for the high satisfaction rates in our series could be:

• We used PP only as a last resort for severe cases where an adequate trial of oral and injectable therapies had failed. Early in our experience we found that the men who were getting full erections with ICI were dissatisfied with SPP since the erection produced by the SPP did not match up to the full erection induced by the ICI. Hence, we did not implant men who were responding well to the ICI. For similar reasons men with short penis (<9 cm) due to loss of length following pelvic fracture and multiple urethroplasties were encouraged to use ICI (if effective) rather than have a PP.
• Extensive counselling on realistic expectations: patients were told to expect that post-implantation the penis would be 10% shorter, narrower and less rigid as compared to their earlier normal erections. SPL was demonstrated in the clinic and used as the bench mark for post-op expectations.
• The limitations of a non-inflatable PP were clearly discussed including the possible need for modification of underwear to achieve concealment.
• The presence of residual penile tumescence (present in 82.7% of patients) following arousal played an important role in improving rigidity and making the erections feel natural during sexual arousal.

An Egyptian study^15^ on SPP satisfaction in the different age groups observed that the EDITS scores of group I (<45 years age), group II (45–65 years) and group III (>65 years) at 12 months were 92.6 ± 5, 90.8 ± 3.7 and 90.2 ± 4.8 respectively (*P* value = .06) showing that the satisfaction of the patients above 65 years of age following Genesis SPP (Coloplast, Minneapolis, MN, USA) were similar to those in less than 65 years of age. Our series also noted that the patients >50 years of age had high satisfaction rates similar to that of patients with <50 years of age with the semi-rigid Shah PP.

In the search for potential predictors for patient dissatisfaction following PP surgery, Akin-Olugbade O et al^16^ reported that the patients with BMI > 30 kg/m^2^ had lower satisfaction rate and had ≥5-point difference in IIEF satisfaction domain scores(OR = 2.2). Our study contradicted the above and noted no such negative effect of higher BMI on patient satisfaction with Shah PP. The mean EDITS score (0–100) were 95.67 ± 10.76, 95.53 ± 8.46 and 91.72 ± 22.42 in patients with BMI <25, 25-29.9 and >30 respectively in our study.

SPP is effective in the management of PD with co-existent ED. Habous M et al observed nearly similar satisfaction rates of 4.4 ± 0.7 and 4.3 ± 0.8 (on a 5-point Likert scale) with SPP and IPP in PD respectively.^17^ Similarly, the 3 patients with PD in our series who received Shah PP reported the maximum overall satisfaction of 4 on a scale 0–4.

A multi-institutional study from Turkey reported that the revision surgery due to corporal perforation was highest in SPP group (2.6%) and device malfunction was highest in 3-piece IPP group (5.5%).^18^ We noted only 1 case of impending corporal perforation which was repaired successfully. Mechanical failures are very rare in SPP^19^ and we saw none in our series as well.

Cayan S et al^18^ reported 4.3 % (15/349) minor complications and 1.1% (4/349) major complications with SPP (AMS Spectra), whereas a large European study by Natali A et al^14^ reported 25% (10/40) major complications with SPP (AMS 600-650). Our study observed minor complications in 21.4% (6/28) and major complications in 10.7% (3/28) patients reiterating the fact that the Shah PP is not only effective but safe as well.

Residual penile tumescence after PP implantation has been reported by Subrini (1982) and is the basis of the soft Subrini PP.^20^ In his series of 283 patients receiving Subrini PP, 68% of the patients had residual penile erection during intercourse thereby enhancing the erection and sexual satisfaction. Similarly, 82.7% of our patients subjectively reported “good” or “some” residual penile tumescence during arousal which improved the overall satisfaction in our series.

The concealment of the SPP is an important aspect which needs special attention. Trivial trauma due to poor concealment of SPP may even lead to significant injuries requiring revision surgeries or device explantation.^21^ With the advantage of soft silicone hinge and careful selection of the correct model of the Shah PP, 90.4% of our patients reported no problem with concealment; however, 1 patient did request explantation due to difficulty with concealment.

The present study has following limitations: partially retrospective nature of the study, recall bias, selection bias, small sample size, absence of a comparison group and part of the follow-up questionnaire was unvalidated. Also, the partner was not interviewed for satisfaction with the PP. But the questions 6 and 7 in our modified EDITS revealed good satisfaction scores (3.76/4) and high partner preference (3.82/4) with respect to the patients’ impression about their respective partners.

### Surgical Pointers

To optimize the results of Shah PP, the following precautions are recommended:

• Position the hinge correctly: select the model appropriate to the SPL (as per Table 1) so that the hinge segment is positioned at the base of the penis with around 2 cm projecting in front of the symphysis pubis.
• Remove sleeves to obtain optimal diameter: there should be a snug fit in the corpora; too narrow an implant will wobble while too wide an implant will risk erosion.
• Segmental sleeve removal: in many men the tips of the corpora taper and are significantly narrower than the main shaft; removal of the distal 2–3 cm of 1 or both sleeves produces an implant that is wide in the shaft, but narrow at the tip. This allows a proper fit in the glans, while allowing a wider diameter PP in the corpus cavernosum.
• Adjust total length correctly: the rear (proximal) end can be trimmed as needed depending on total corporal length and appropriate RTE may be added.

In difficult cases the Shah PP allows the following additional maneuvers:

• In the event of corporal perforation, the PP should be positioned correctly and then fixed to the tunica with a suture that passes through the PP and the tunica. This can be done easily since there is no metal wire in the implant and will prevent the PP from migrating while the perforation is healing.
• In patients with extensive corporal fibrosis, there may be a segment of the corpus that cannot be dilated adequately. The Shah PP can be selectively shaved to reduce the diameter of the corresponding segment of PP so that the corpora can be closed without the need for a mesh.

Current practice trends favour implantation of IPP because of its superior functioning. However, in developing countries, where PP are not covered by insurance and per capita income is often less than USD 500 per month, patients cannot afford expensive IPP and even standard SPP from international companies are beyond the reach of most. Hence, there is a large population of men with ED whose needs remain unserved. The low cost of the Shah PP make it ideal for patients with refractory ED in low income countries, and also make it suitable for cases of salvage procedures (infection or priapism) and in men with poor dexterity.

## CONCLUSION

Shah PP is a low-cost SPP which offers good satisfaction with minimal complications in properly selected and adequately counseled men with refractory ED. The option to remove 1 or 2 sleeves to change the diameter of the PP is extremely helpful in achieving a snug fit with a small inventory, and the soft silicone hinge has yielded very good concealment in nearly 91% of the patients.

## STATEMENT OF AUTHORSHIP

Conceptualization, P. Krishnappa, A. Tripathi, R. Shah; Methodology, P. Krishnappa, A. Tripathi, R. Shah; Investigation, P. Krishnappa, A. Tripathi; Writing – Original Draft, P. Krishnappa; Writing – Review & Editing, P. Krishnappa, A. Tripathi, R. Shah; Supervision, P. Krishnappa, A. Tripathi, R. Shah.

## Appendix Supplementary materials

Image, application 1

## Footnotes

Supplementary material associated with this article can be found in the online version at doi:10.1016/j.esxm.2021.100399.
