# Supplementary material for: Surgical Outcomes and Patient Satisfaction With the Low-Cost, Semi-Rigid Shah Penile Prosthesis: A boon to the Developing Countries
Source: Sex Med. 2021 Jul 16;9(4):100399. doi: 10.1016/j.esxm.2021.100399 (PMC8360909; doi:10.1016/j.esxm.2021.100399)
Supplement: Supplementary file 1 [file mmc1.pdf]

Supplementary file

Modified EDITS patient`s survey form (English)

The questions in this inventory ask about a sensitive topic, your sexual life with your wife or partner as well as your attitude toward and expectations from the penile prosthesis to help you with your erection problem.

Please answer the questions as honestly and candidly as you can. If any questions or terms are unclear, please ask for clarification.

1. Overall, how satisfied are you with penile prosthesis?		SCORE
a.Very satisfied	4	
b.Somewhat satisfied	3	
c.Neither satisfied nor dissatisfied	2	
d.Somewhat dissatisfied	1	
e.Very dissatisfied	0	
2. To what degree has penile prosthesis met your expectations?		
a. Completely	4	
b. Considerably	3	
c. Half way	2	
d. A little	1	
e. Not at all	0	
3. How likely are you to continue using penile prosthesis?		
a. Very likely	4	
b. Moderately likely	3	
c. Neither likely nor unlikely	2	
d. Moderately unlikely	1	
e. Very unlikely	0	
4. How easy was it for you to use penile prosthesis?		
a. Very easy	4	
b. Moderately easy	3	
c. Neither easy nor difficult	2	
d. Moderately difficult	1	
e. Very difficult	0	
5.How confident has penile prosthesis made you feel about your ability to engage in sexual activity?		
a. Very confident	4	
b. Somewhat confident	3	
c. It has had no impact	2	
d. Somewhat less confident	1	

For Review Only

e. Very much less confident	0	
6. Overall, how satisfied do you believe your partner is with the effects of penile prosthesis?		
a. Very satisfied	4	
b. Somewhat satisfied	3	
c. Neither satisfied nor dissatisfied	2	
d. Somewhat dissatisfied	1	
e. Very dissatisfied	0	
7. How does your partner feel about your continuing to use penile prosthesis?		
a. My partner absolutely wants me to continue	4	
b. My partner generally prefers me to continue	3	
c. My partner has no opinion	2	
d. My partner generally prefers me to stop	1	
e. My partner absolutely wants me to stop	0	
8.How natural did the process of achieving an erection feel with penile prosthesis over the past four weeks?		
a. Very natural	4	
b. Somewhat natural	3	
c. Neither natural nor unnatural	2	
d. Somewhat unnatural	1	
e. Very unnatural	0	
9.Compared with before you had erection problem, how would you rate the naturalness of your erection with penile prosthesis over the past four weeks in terms of hardness?		
a. A lot harder than before I had an erection problem	4	
b. Somewhat harder than before I had an erection problem	3	
c. The same hardness as before I had an erection problem	2	
d. Somewhat less hard than before I had an erection problem	1	
e. A lot less hard than before I had an erection problem	0	
10. How satisfied are you with the appearance of your penis in resting state?		
a. Very satisfied	4	
b. Somewhat satisfied	3	
c. Neither satisfied nor dissatisfied	2	
d. Somewhat dissatisfied	1	
e. Very dissatisfied	0	
11. How satisfied are you with the concealment of your penile prosthesis?		
a. No problem at all	4	
a. Concealment possible with modification of underwear	3	
b. Some difficulty despite all measures	2	
c. Significantly awkward	1	
d. Very dissatisfied	0	

For Review Only

Total EDITS (Max 44):
Mean EDITS = Total EDITS / 11
Mean EDITS score = mean EDITS x 25

For Review Only
